# Discovery of a New Biomarker Pattern for Differential Diagnosis of Acute Ischemic Stroke Using Targeted Metabolomics

**DOI:** 10.3389/fneur.2019.01011

**Published:** 2019-09-19

**Authors:** Ruitan Sun, Yan Li, Ming Cai, Yunfeng Cao, Xiangyu Piao

**Affiliations:** ^1^Department of Neurology, Affiliated Zhongshan Hospital of Dalian University, Dalian, China; ^2^Department of Instrumentation and Analytical Chemistry, Dalian Institute of Chemical Physics, Dalian, China

**Keywords:** metabolomics, acute ischemic stroke, biomarkers, mass spectrometry, amino acids, carnitine

## Abstract

Stroke is one of the leading causes of disability all over the world. However, biomarkers for fast differential diagnosis of acute ischemic stroke (AIS) from vertigo or headache, remains lacking. Using a direct-infusion mass spectrometry method, it is possible to establish an efficient method for AIS differential diagnosis that requires only a few minutes. Thirty-eight clearly diagnosed AIS patients and 46 patients with a main complaint of vertigo were enrolled in this study. There was a total of 58 metabolites that were measured by our targeted metabolomics method, and the data were analyzed by pattern recognition algorithms. As a result, a clear classification between AIS and vertigo patients was achieved. Acylcarnitines are the major discriminating metabolites between the two groups. Arginine and its ratio, which is related to urea cycle metabolites, including arginine/ornithine and citrulline/arginine, also accounted for the classification. Interestingly, the levels of these metabolites were also found to be restored among recovering AIS patients (*n* = 11), which indicated that the metabolic alterations are possibly related to AIS development. Based on the characters from the data pattern reorganization, a novel biomarkers pattern was established using a binary logistic model, which contained arginine, arginine/ornithine, vaccenylcarnitine, and hydroxylbutyrylcarnitine. This biomarkers pattern achieved an area under the receiver operating characteristic curve of 0.89 for the differential diagnosis of AIS. Considering the efficiency and the diagnostic performance of the biomarkers pattern, our method has potential future use for the clinical application.

## Introduction

Stroke is the second most common cause of disability worldwide, accounting for ~9% of deaths every year (1). It is also a serious health problem in China, where there are ~1 million new patients that are diagnosed with stroke each year (2), a number that is also constantly increasing. Stroke is typically classified into two basic subtypes: ischemic and hemorrhagic. Approximately 80% of strokes are ischemic, and are caused by a bloodstream blockage that leads to brain tissues ischemic damage (3). Computed tomography (CT) and magnetic resonance imaging (MRI) scans are common tools for stroke diagnosis. Although imaging diagnosis can provide direct clinical evidence of stroke, these scans are still time-consuming, which may delay the therapeutic window for thrombolytic therapy following a stroke by 3–4.5 h (4). In addition, it is difficult to differentiate the clinical symptoms of an acute ischemic stroke (AIS) from transient ischemic attacks (TIAs), that may also exhibit tissue lesions on MRI images (4). Therefore, it is necessary to identify novel biomarkers for AIS that can clinically provide efficient analytical tools.

Metabolomics are considered a powerful tool for the classification of diseases and the discovery of new biomarkers from a pool of small molecules (5). Previous metabolomic studies investigated the use of metabolic biomarkers for the diagnosis or the investigation of the pathological mechanisms of stroke using untargeted metabolomics to determine the metabolic features of ischemic stroke (IS) (6), amino acid signatures (7), AIS progression (8), and TIA differential diagnosis (9). Some metabolites were found to be related to AIS occurrence and development and have been studied by targeted metabolomics methods. These studies included the diagnosis of post-stroke cognitive impairment (10), the relationship between lysine and high-risk stroke patients (11). Considering the efficiency and high-throughput nature of the metabolomic methods, the typical liquid chromatography, coupled to mass spectrometry (LC/MS)- or gas chromatography (GC)/MS-based metabolomics platforms, is not suitable for fast diagnosis, due to the complicated and time-consuming procedures for sample collection, pre-treatment, and chromatographic analysis. Direct infusion mass spectrometry is a high-throughput method of targeted or untargeted analyses that can be performed within 1–2 min. Thus, it is possible to use this method to develop novel diagnostic panels for the fast identification of AIS in patients with the chief complaint of headache or vertigo. In this study, we present a metabolomics approach that is based on an LC/MS direct infusion method to identify a potential biomarker panel for the fast diagnosis of AIS and to differentiate it from other cerebral diseases.

## Materials and Methods

### Patients

Patients and controls were hospitalized in the Affiliated Zhongshan Hospital of Dalian University. The study was approved by the ethics committee of the hospital. Written informed consents were given by all the participants. AIS diagnosis in patients (*n* = 38) was based on the *Chinese guidelines for diagnosis and treatment of acute ischemic stroke 2018* (12). Forty-six control patients were also enrolled with the chief complaint of headache or vertigo, and no ischemic lesions were found among these patients. The results of CT and MRI confirmed the diagnosis of AIS and the controls. The AIS group consisted of 10 females and 28 males, and the control group consisted of 18 females and 28 males. The average age of the AIS and the control groups was 67.34 years (50–89 years) and 66.06 years (54–85 years), respectively. No statistical significance in gender (chi-squared test, *p* = 0.11) or age (Student's *t*-test, *p* = 0.36) was found between the two groups. The blood sugar levels in the AIS and control groups were 5.8 ± 1.4 and 5.3 ± 0.9 mmol/L, respectively. The concentrations of triglyceride were 1.2 ± 0.6 mmol/L in the AIS group, and 1.1 ± 0.4 mmol/L in the control group. No statistical significance was found between the two groups in the above clinical tests (Student's *t*-test, *p* > 0.05).

For the inclusion criteria, all AIS patients and controls were 50–89 years old. No statistical significances in gender and age were found between the two groups. Individuals with malignancies, infectious, autoimmune, and cardiovascular diseases were excluded from this study.

Blood was collected from fasting patients on the first morning in the hospital. Whole blood was dropped on a DBS paper and immediately stored at −80°C until analysis.

### Chemicals

Acetonitrile was purchased from Thermo Fisher Scientific (Waltham, MA). Acetyl chloride, 1-butanol, amino acids, and acylcarnitine reference standards, were purchased from Sigma-Aldrich (St. Louis, MO). The water was filtered using a Milli-Q system (Millipore, MA). The stable isotope-labeled amino acids and acylcarnitines were purchased from Cambridge Isotope Laboratories (Tewksbury, MA).

### Sample Preparation

Metabolites extraction from DBS samples has previously been described (13). Briefly, DBS paper cut to a diameter of 3 mm was placed into a Millipore MultiScreen-HV 96-well plate (Millipore, Billerica, MA) and a, 100 μl of working solution was added for metabolite extraction. After shaking for 20 min and centrifugation at 1,500 × g for 2 min, the filtrates were transferred to new tubes and dried under nitrogen at 50°C. The samples were derivatized with 60 μl acetyl chloride/1-butanol (10:90, v/v) mixture at 65°C for 20 min, dried under nitrogen at 50°C and reconstituted in 100 μl of 80% acetonitrile/water (v/v) before analysis.

### Targeted Metabolic Analysis

A flow injection method was used for the targeted analysis. A 20 μl sample was injected with an initial flow rate of 0.2 μl/min. The flow rate was dropped to 0.01 μl/min and held at this rate for 1.5 min before increasing and maintaining the rate at 0.2 μl/min for 0.5 min. An AB Sciex 4000 QTRAP system (AB Sciex, Framingham, MA, USA) was used for the targeted analysis. The samples were analyzed in ESI+ mode. The curtain gas pressure was 20 psi and the pressure of gas 1 and gas 2 was set at 35 psi. A 4.5 kV ion spray voltage was used and a 350°C auxiliary gas temperature was maintained. The ion pairs for the amino acids and acylcarnitines were described in our previous work (13).

### Data Analysis

The raw data were collected with the Analyst v1.6.0 software (AB Sciex) and processed by the ChemoView 2.0.2 (AB Sciex). The data output was transferred into Excel format for further analysis. The multivariate data analysis, including the principal component analysis (PCA), and the partial least squares discriminant analysis (PLS-DA), was carried out with the SIMCA-P (version 13.0, Umetrics, Sweden). The statistical analysis, including Chi-square test and Student's *t*-test, was performed with IBM SPSS Statistics 18.0 software. The cut-off alpha value was set at 0.05.

## Results

### Targeted Metabolite Profiling of AIS Patients

**Figure 1:**
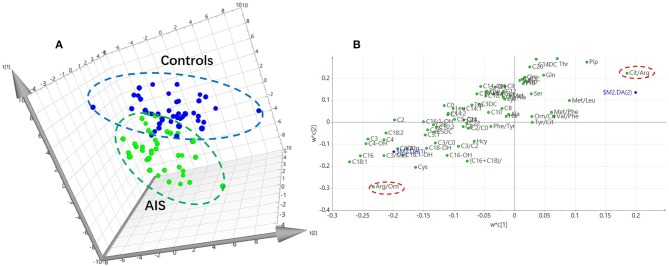
PLS-DA analysis of the metabolomics data (*R*^2^*Y* = 0.61, *Q*^2^ = 0.23). **(A)** PLS-DA score plot. Acute ischemic stroke (AIS) and the controls can be discriminant on the 3D score plot. **(B)** Loading plot of the PLS-DA analysis. The impact of the metabolites on the classification could be seen on the plot.

**Figure 2:**
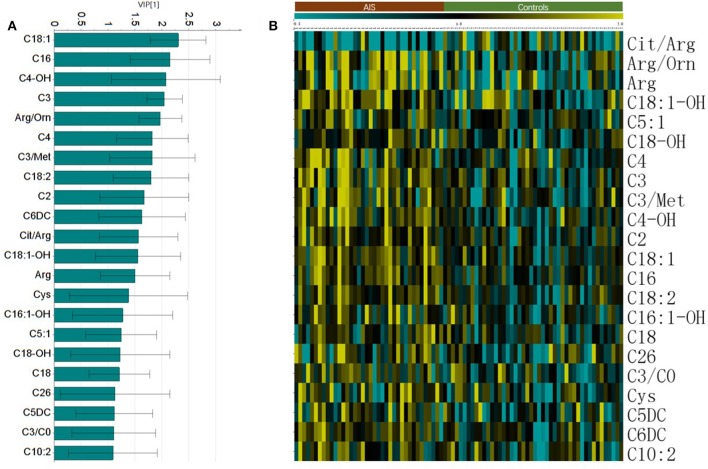
The important metabolites for the classification of AIS. **(A)** Metabolites with Variable importance in the project (VIP) larger than one. **(B)** Heatmap of the significant changed metabolites (student's *t*-test, *p* < 0.05).

In this study, an LC/MS-based targeted metabolomics approach was used to quantify 23 amino acids and 35 carnitines. Moreover, 14 ratios between the quantified metabolites were also calculated and that reflects the relative abundance of related enzymes (Table 1). To find potential AIS biomarkers, PLS-DA analysis was performed with the SIMCA-P software (*R*^2^*Y* = 0.61, *Q*^2^ = 0.23). No overfitting was found according to the results of the permutation test (*R*^2^ intercept is 0.37, *Q*^2^ intercept is −0.32). As observed on the PLS-DA score plot in Figure 1A, AIS and control patients are clearly distinguishable on the plot. Potential biomarkers for the diagnosis of AIS could be seen from the loading plot (Figure 1B). Arginine/ornithine (Arg/Orn) and citrulline/arginine (Cit/Arg) had the most important differential metabolite ratios from the PLS-DA model. Some acylcarnitines, such as vaccenylcarnitine (C18:1), palmitoylcarnitine (C16), and 3-hydroxylbutyrylcarnitine (C4OH), were also core metabolites for the classification of AIS and vertigo patients. VIP (variable importance in the project) scores were used to determine potential biomarkers for diagnosis. The first 22 biomarkers are listed in Figure 2A and have VIP scores larger than 1. Arg and its Arg/Orn and Cit/Arg related ratios were recognized as candidate markers for AIS. Acylcarnitines with short c (*n* < 5) and long carbon chains (*n* > 16) were also included in the list.

**Table 1:** The concentrations of significantly differential metabolites.

**Metabolites/ratios**	**AIS**		**Controls**			**Fold change**	**Regulation**
	**Mean**	**SD**	**Mean**	**SD**	***p*-value**		
Arg	8.40	4.88	6.96	3.26	3.1E-03	1.21	↑
Cys	1.56	1.17	0.80	0.47	6.6E-03	1.95	↑
C2	13.29	10.44	4.51	3.02	8.8E-04	2.95	↑
C3	1.43	1.01	0.51	0.36	3.5E-05	2.79	↑
C4	0.22	0.16	0.09	0.05	2.6E-04	2.51	↑
C4-OH	0.10	0.07	0.04	0.02	2.6E-05	2.39	↑
C5DC	0.10	0.08	0.05	0.04	2.9E-02	1.82	↑
C5:1	0.06	0.05	0.02	0.02	1.5E-02	2.79	↑
C16	1.14	0.85	0.33	0.22	1.2E-05	3.39	↑
C16:1-OH	0.07	0.06	0.02	0.02	1.3E-02	2.74	↑
C18	0.51	0.44	0.16	0.13	1.8E-02	3.28	↑
C26	0.05	0.04	0.02	0.02	2.7E-02	2.29	↑
C18:1	0.82	0.60	0.23	0.17	2.2E-06	3.62	↑
C18-OH	0.03	0.02	0.01	0.01	1.6E-02	3.24	↑
C18:1-OH	0.03	0.02	0.01	0.01	2.0E-03	2.47	↑
C10:2	0.86	0.71	0.30	0.31	3.3E-02	2.87	↑
C18:2	1.90	1.48	0.59	0.44	3.3E-04	3.20	↑
C6DC	0.89	0.71	0.24	0.26	1.2E-03	3.71	↑
Arg/Orn	0.44	0.27	0.25	0.12	7.0E-05	1.79	↑
Cit/Arg	3.58	5.38	2.41	2.67	1.9E-03	1.49	↑
C3/C0	0.05	0.04	0.01	0.02	3.1E-02	3.04	↑
C3/Met	0.08	0.06	0.03	0.02	2.7E-04	2.40	↑

*Arg, Arginine; Cit, Citrulline; Met, Methionine; Orn, Ornithine; C, carnitine. (↑), Upregulated; (↓), downregulated. The cut-off alpha value was 0.05 for student's t-test*.

### Statistical Analysis of the Data

**Figure 3:**
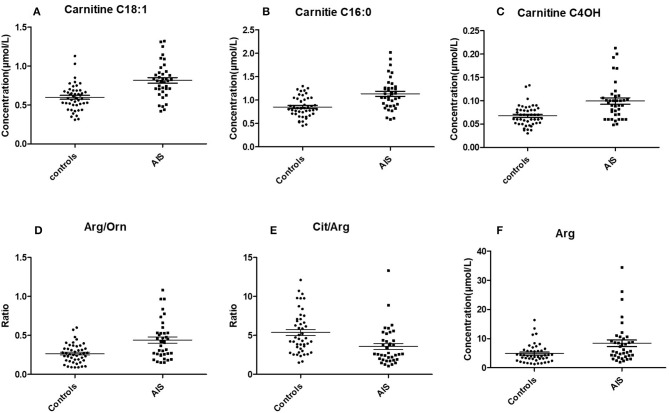
Box plot of the potential markers for the diagnosis of AIS (mean ± SEM). **(A)** Vaccenylcarnitine (carnitine C18:1), **(B)** Palmitoylcarnitine (carnitine C16:0), **(C)** 3-Hydroxylbutyrylcarnitine (carnitine C4 OH), **(D)** arginine/ornithine, (Arg/Orn), **(E)** citrulline/argigine (Cit/Arg). **(F)** Arginine (Arg).

The Student's *t*-test was used to find and confirm the potential biomarkers from the data. There were 22 significantly changed variables (metabolites and their corresponding ratios) in the dataset (*p* < 0.05). The relative levels (normalized to controls average) of these variables can be seen on the heat map (Figure 2B). The clustering of these metabolites according to the Pearson correlations represents a possible relationship among these potential biomarkers. On the heat map, the level of Cit/Arg was decreased in the AIS group compared with that in the control group. Arg, Arg/Orn and acylcarnitine levels were increased in the AIS group compared to those in the control group. A box plot of the differential metabolites can be seen in Figure 3, which includes vaccenylcarnitine (C18:1), palmitoylcarnitine (C16:0), 3-hydroxylbutyrylcarnitine (C_4_OH), arginine/ornithine (Arg/Orn), citrulline/arginine (Cit/Arg), and arginine (Arg). These metabolites and their corresponding ratios showed deregulations of Arg and acylcarnitine metabolisms during AIS.

### Establishment of a Diagnostic Model for AIS

**Figure 4:**
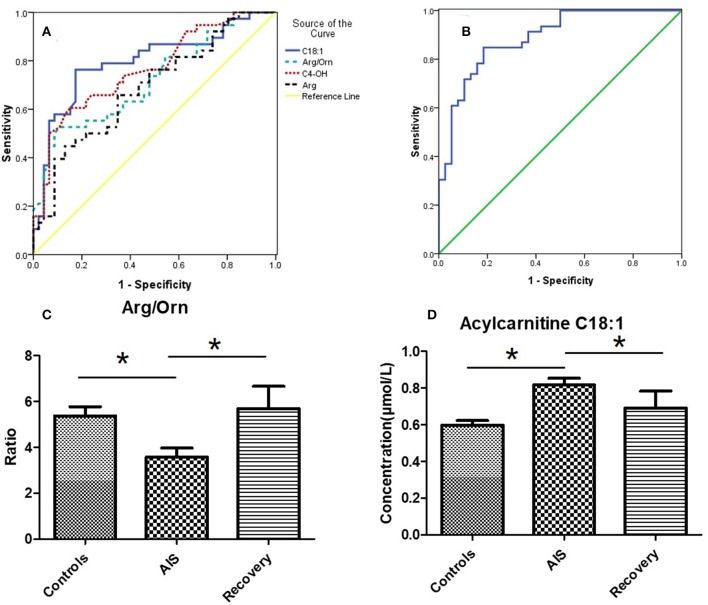
**(A)** Receiver operating characteristic curve (ROC) of 4 potential markers. The area under the curve (AUC) of Carnitine C18:1 is 0.80, the AUC of Arg/Orn is 0.72, the AUC of C4OH is 0.77, the AUC of Arg is 0.69. **(B)** ROC of the combination of 4 markers (mean + SEM). The AUC is 0.89. **(C,D)** The concentrations of Arg/Orn and carnitine C18:1 in controls group, AIS group and recovery group. *p < 0.05, student's *t*-test.

Based on the potential metabolites that were selected above, we established diagnostic models using all the significantly changed metabolites. A binary logistic regression model was used to establish the diagnostic model for AIS, and the diagnostic performances were evaluated by a receiver operating characteristic curve (ROC, Figures 4A,B). The area under the curve (AUC) was 0.80, for carnitine C18:1, 0.72 for Arg/Orn, 0.77 for C_4_OH, and 0.69 for Arg. When these four variables were combined to set up the diagnostic model, the AUC was 0.89.

To validate the results of the biomarker discovery, 11 IS recovering patients were enrolled in the study. Samples were collected 8 days after the occurrence of ischemic stroke. As shown in Figures 4C,D, the ratio of Arg/Orn increased to a normal level following IS. The concentration of acylcarnitines (e.g., carnitine C18:1) decreased to a normal level.

## Discussion

Headache and vertigo are common clinical symptoms that are due to a diverse set of causes. Therefore, the differential diagnosis of these diseases is rather complicated without direct evidence from CT or MRI inspections. Among all patients with headaches or vertigo, AIS is one of the most urgent diseases that requires a rapid response and there are only a few hours within the narrow therapeutic window for thrombolytic therapy. Therefore, we attempted to establish a fast-diagnostic method that is based on metabolomic data. Most of the current metabolomics studies try to discover novel biomarkers for stroke by comparing patients and healthy controls. In this study, we paid more attention to the biomarkers that allow differential diagnosis of AIS from patients with headaches or vertigo. In addition, direct infusion MS ensures the efficiency of the method that considers the urgency of AIS. After a routine sample collection procedure, 23 amino acids and 35 carnitines from DBS samples were measured within 2 min, making this method a great tool for fast AIS clinical diagnosis.

To gain more information about the metabolites related enzymes, 12 ratios were calculated and used as variables for data analysis. The metabolites and their corresponding ratios can be referred to in our previous work (13). From the results of the multivariable analysis (PLS-DA), potential biomarkers were found for the differential diagnosis of AIS and vertigo. The results showed that carnitines and arginine may be core metabolites for the diagnosis of AIS and vertigo in patients (Figure 1B). Metabolites that are associated with the arginine synthesis pathway, including Orn and Cit, could also be dysregulated in AIS patients. Arg is a substrate for the biosynthesis of nitric oxide (NO), a well-known endogenous vasodilator (14). The increased level of Arg during AIS may indicate an upregulated NO biosynthesis with feedback from the brain anaerobic state. Moreover, Arg and its related metabolites, such as asymmetric dimethylarginine and guanidinoacetate, have been shown to be potential biomarkers for cardiovascular diseases that are related to aortic atherosclerosis (15). The increase in the Arg level and its corresponding ratios (Cit/Arg and Arg/Orn) may reflect abnormal metabolism in the pathway, which could result from artery obstruction. Acylcarnitines are the intermediates of fatty acid β-oxidation and transport fatty acids through the mitochondria membranes. The increased levels of acylcarnitines among AIS patients may indicate an increased energy requirement due to a negative feedback from the brain hypoxic state (16). Acylcarnitines are also related to the recovery of stroke (17), and Arg and carnitines have been reported as important metabolites associated with ischemic strokes (18). In fact, many amino acids have been reported to be related to ischemic stroke occurrence (19). Metabolites including branch-chained amino acids (BCAA), homocysteine, and metabolites in the folate cycle has been shown to be associated with different stroke stages (19). Homocysteine (Hcy) has been considered as an independent human stroke risk factor (20). BCAAs are also considered risk factors for stroke and other cerebrovascular diseases (21) and elevated levels of serum free fatty acids may be related to the outcome of AIS (22). These studies demonstrates the deregulation state when stroke occurs.

In our study, we focused on the differential diagnosis among patients that complained of headache or vertigo. In the early stage of AIS, we did not find differences between AIS and other headache diagnosed patients for some metabolic markers, such as Hcy. Those metabolic deregulations may not be accumulated to a significant level. Fortunately, Arg and acylcarnitines are found to be closely related to the acute state of the ischemic stroke attack. The results from recovering AIS patients are also consistent with these observations. The levels of Arg and carnitines returned to normal 8 days after the attack. Changes in the levels of Arg and carnitines are thought to result from elevated angiectasis and an increase in the energy supply of the body

The use of all significant metabolites changes to construct the diagnostic model seems promising and could clearly classify AIS and control patients (AUC = 1). However, a diagnostic model containing 22 variables is too complicated for clinical use and to simplify the diagnostic model, we constructed a biomarkers pattern according to the results of PLS-DA. Therefore, the levels of arginine, its related ratios, and carnitines were used, and showed an AUC of 0.89. The sensitivity and specificity of this pattern is acceptable for a fast diagnosis of AIS.

We also analyzed samples from patients who survived after the acute stage (8 days after AIS) and found that the ratio of Arg/Orn reached the normal level that is seen in the controls (Figure 4C). The arginine content also decreased to normal levels (data not shown). In addition, the concentrations of carnitines were also restored to normal levels. These results indicate the body's recovery from acute cerebral hypoxia.

In summary, a targeted metabolomics method was used for the early differential diagnosis of AIS. A serological biomarkers pattern was established with a quick analytical technique using DBS and direct infusion. Based on our preliminary results, these metabolic markers are helpful for the early differential diagnosis of AIS from patients with vertigo. However, a multicenter trial with large scale samples is required for validation and it is also important to use other molecular biomarkers, such as lipids or proteins, which may improve the sensitivity and specificity of the biomarkers pattern. We believe that such molecular markers have great potential for further clinical applications.

## Data Availability Statement

This manuscript contains previously unpublished data. The name of the repository and accession number(s) are not available.

## Ethics Statement

This studies involving human participants were reviewed and approved by the Affiliated Zhongshan Hospital of Dalian University. The patients/participants provided their written informed consent to participate in this study. Written informed consent was obtained from the individual(s) for the publication of any potentially identifiable images or data included in this article.

## Author Contributions

RS: design the study, analyze the data, and write the paper. YL: study the patients and analyze the data. MC: provide discussion of the data and the diagnosis of the patients. YC: analyze the data using manuscript. XP: design the study and discuss the results.

### Conflict of Interest

The authors declare that the research was conducted in the absence of any commercial or financial relationships that could be construed as a potential conflict of interest.
